# Perception of falsified and counterfeit medicines among adults living in Mexico City and the metropolitan area: an underappreciated health risk

**DOI:** 10.3389/fphar.2025.1654822

**Published:** 2025-08-29

**Authors:** Jessica Citlalli Celaya Pérez, Tania Ibargüengoitia Larios, Maria de los Angeles Frías Fernández, Manuel Abraham Gómez Martínez, Kenneth Rubio Carrasco, Raúl Lugo Villegas, Arely Vergara-Castañeda

**Affiliations:** ^1^ Facultad de Ciencias Químicas, Universidad La Salle México, Mexico City, Mexico; ^2^ Programa jóvenes a la investigación en matemáticas y ciencias experimentales. Escuela Nacional Colegio de Ciencias y Humanidades plantel Azcapotzalco, Universidad Nacional Autónoma de México, Mexico City, Mexico; ^3^ Departamento de Salud Pública, Facultad de Medicina, Universidad Nacional Autónoma de México, Mexico City, Mexico; ^4^ Facultad de Química, Universidad Nacional Autónoma de México, Mexico City, Mexico; ^5^ Vicerrectoría de Investigación, Universidad La Salle México, Mexico City, Mexico

**Keywords:** falsified drugs, health risk, adults, public health, pharmacoepidemiology, epidemiology

## Abstract

Falsified and counterfeit medicines represent a growing global public health concern and straddles business implications, and their proliferation has intensified worldwide. An analytical cross-sectional study was carried out to validate and assess the perception of a group of residents of Mexico City and the metropolitan area regarding the problem of falsified drugs and the risks they entail, along with the purchase of medicines at unauthorized points of sale. In addition, variables associated with buying or not buying at these points of sale were identified. The results suggest that even in a sample of mainly young adults, the potential exposition to collateral effects related to the consumption of falsified or counterfeit medicines is high. Although the participants recognized that these medicines are not safe, they are not familiar with the efforts or information of the corresponding authority to warn them of this problem. In addition, low awareness of the real implications of their nature and their possible impact on heath was identified, suggesting that the falsified and counterfeit medicines’ market is a substantial and understudied issue among the Mexican population.

## 1 Introduction

Nowadays, falsified and counterfeit medicines represent a growing global public health concern and straddles business implications, and its proliferation has intensified (A Ziavrou et al., 2022); they are considered a more significant public health threat than diseases they purport to cure (McManus and Naughton, 2020; Ofori-Parku and Park, 2022). Even though the World Health Organization (WHO) defines a falsified drug as “a product that is deliberately and fraudulently mislabeled as to its identity, composition, and/or source” (World Health Organization, 2010a), the European Medicines Agency distinguishes falsified drugs from the counterfeit ones as those “designed to mimic real medicines” and meanwhile considers counterfeit medicines as “medicines that do not comply with the intellectual property rights” (Feeney et al., 2024). Furthermore, in the case of Mexico, the national regulatory agency of the Ministry of Health, the Federal Commission for the Protection against Sanitary Risks (its acronym in Spanish COFEPRIS), considers falsified medicines to be a health risk as they could be made with contaminated, toxic substances or, in some cases, lose effectiveness due to incorrect production, distribution, and storage conditions (Comisión de Fomento Sanitario, 2022).

These subtle differences and the lack of a definition with consensus hinders discussions regarding this global health issue, which can affect both patent and generic medicines, with serious consequences beyond individual harm, affecting the therapeutic efficacy and safety of the patient who consumes them by increasing the risk of treatment failures, adverse reactions, mismanagement of their diseases, and even death (Bottoni and Caroli, 2019), along with exacerbating drug resistance, developing antimicrobial resistance, compromising disease control efforts, undermining public trust in healthcare systems and their regulatory practices by wasting resources (Feeney et al., 2024), and significant economic implications, which could increase the vulnerability of the people of the most unprotected sectors, who generally do not have access to health services and lack information to identify falsified drugs (Comisión de Fomento Sanitario, 2022).

The WHO has pointed out that one of 10 medicines in Mexico and the region is falsified or of low quality (González, 2024). In addition, according to COFEPRIS, the number of types of falsified drugs has increased by up to 300% of what was reported in 2019 compared to the recent figures from 2022 (Rodríguez, 2024); in addition, the report by the Mexican Association of Pharmaceutical Research Industries (AMIF) suggested that 60% of the medicines marketed in the country are stolen, expired, falsified, or produced without the minimum quality requirements (Meraz, 2018). Throughout this year, COFEPRIS has issued approximately 62 health alerts, of which approximately 20 have been regarding falsified drugs (Supplementary Appendix A1; Supplementary Appendix Table 1A); from this list, the following drugs that are quite commonly used by Mexican population can be highlighted, which include aspirin^®^, desenfriol^®^, tabcin^®^, eutirox^®^, dolo-neurobión^®^, and lakesia^®^, among others (Secretaria de Salud del Estado de Nuevo León, 2024).

Although individual studies have highlighted the general relevant aspects related to this issue, most of them are mainly focused on the economic impact, regulatory or normative factors, health impact, and technologies to address this challenge, and only a few of them assessed the consumers’ attitudes, risk perceptions, and purchase intentions (Secretaria de Salud del Estado de Nuevo León, 2024). As a result, the first step to counteract this problem efficiently is to identify the areas or information to be disseminated and reinforced to promote an adequate culture of the use of medicines, and the risks involved in the purchase and use of falsified drugs, particularly at unauthorized points of sale (Comisión de Fomento Sanitario, 2022; Protección Contra Riesgos Sanitarios, 2013). Given the potential public health risk associated with falsified and counterfeit medicines, the main objective of this study was to assess the perception and awareness of the perceived risks that they entail while also considering the potential consumers’ knowledge and decision-making among a group of residents of an urban area in Mexico.

## 2 Materials and methods

This cross-sectional study comprised two phases; in the first one, a cultural design and validation assessment through a self-administered questionnaire was performed, considering the content, construction, and criterion validity, along with a feasibility analysis. In the second phase, a preliminary report regarding the perception of falsified and counterfeit medicines was integrated among a pilot sample.

To create the desired questionnaire, a review of published papers aiming to explore the elements considered to assess the perception of the population regarding the sale and use of falsified medications was performed. Searching included the keywords and Boolean operators “falsified OR counterfeit,” “medicines OR drugs,” and “perception,” Based on the instruments used in these studies, an authorial questionnaire was designed and validated (face and content validity) by the research team, which included the university research workers and independent consultants employed in contract research organizations. In addition, linguistic and grammatical assessment was performed.

In the instrument, 40 items were organized into three sections. After obtaining participants’ informed consent to respond and to allow the use of their responses for research purposes, the following 12 items were aimed at identifying and characterizing the participants (age, gender, occupation, clinical history, and purchase and use of medications within the past 3 months).

The other section considered 16 items aimed to assess the consumer’s knowledge and awareness of falsified and counterfeit medications as a health risk, of which, 1 question included an image of the latest health alert reported by COFEPRIS on this topic to know if this information was recognized by potential end users in the community, 2 more questions aimed to evaluate the perceived direct impact on this topic, and the rest13 questions were posed with six answers on Likert’s scale (I … completely agree/ agree/ neither agree or disagree / disagree / completely disagree) focused on the context of understanding the characteristics of these unregulated medicines (regarding dose, content, contamination or expiration, as well as the possible impact on their health if consumed), and on the other hand, to recognize their abilities to avoid and discern these products from the original ones.

Furthermore, the last 12 items aimed at identifying the factors associated with the intention or possibility of purchasing falsified or counterfeit medicines if necessary. Those dichotomous and ordinal responses were coded, and the open questions were categorized. Descriptive statistics were performed using SPSS IBM version 22.0^®^, and categorical variables are presented as proportions.

Regarding the instrument’s validity, construct validation was performed using exploratory factor analysis (EFA) using principal components, the Promax rotation method with Kaiser normalization, and the assumptions for applying factor analysis were verified using the Kaiser–Meyer–Olkin (KMO) index and Bartlett’s test of sphericity. In addition, a reliability analysis with Cronbach’s α based on standardized elements was performed for all items and each created dimension.

## 3 Results

### 3.1 Characteristics of the participants and the recent consumption/purchase of drugs

#### 3.1.1 Population context

In the preliminary assessment, 97 responses were obtained, of which eight were eliminated because the place of residence was outside Mexico City and the metropolitan area corroborated by the zip code; hence, 89 valid responses were considered for this analysis, of which 55% (n = 49) were women and 45% (n = 40) were men.

#### 3.1.2 Health profile of the respondents

The profile of the participants suggests a young and relatively healthy population, with an average age of 34.9 ± 14.4 years; 69.7% of the respondents reported being workers or employees, whereas 18.0% were students, 6.9% reported other types of occupation, and only 5.6% were exclusively dedicated to the home. Of the participants, 27.0% reported having at least one diagnosed disease, of which the most common were hypothyroidism (29.2%), high blood pressure (20.8%), anxiety (12.5%), dyslipidemia (12.5%), polycystic ovary syndrome (8.3%), diabetes (8.3%), and other diseases, including the following: allergy, depression, epilepsy, migraine, rhinitis, arthritis, acne, gastrointestinal diseases, immune system diseases, and use of hormone therapy (4.2%). No differences were found according to gender (p = 0.142) or occupation, nor related to health activities (p = 0.260).

Regarding the use of medicines in the last 3 months, the consumption of 127 medicines were reported, of which 82.1% were purchased prescribed medicines; meanwhile, 6.7% were remaining medicines from previous treatments without recent purchases, and finally, 2.24% were reported as donations, that is, those medications that were no longer used by other people and were given away to help those who need them. No differences were found between previously diagnosed or apparently healthy individuals (p = 0.166).

The medicines that the participants acquired the most in the last 3 months are described in Table 1, highlighting the purchase of analgesics, drugs for gastrointestinal problems, antibiotics, and anti-flu medications, which suggests treatments for acute conditions. On the other hand, 12.6% of the purchased medicines were controlled drugs sold only with a prescription, whereas the remaining 87.4% were over-the-counter medicines.

**TABLE 1:** Type of medicines purchased by the participants in the study according to the reported use.

Drug category	Proportion according to all purchased medicines in the last 3 months (%), n = 127	Drug category	Proportion according to all purchased medicines in the last 3 months (%), n = 127
Analgesics	70.1 (89)	Ophthalmic	4.72 (6)
Gastrointestinal	16.5 (21)	Pain, fever, or swelling (salicylates)	3.93 (5)
Antibiotics	7.9 (10)	Dyslipidemia	3.93 (5)
Anti-flu	7.1 (9)	Other (multivitamins and probiotics)	3.14 (4)
Hormonal	6.3 (8)	Insulin and antidiabetics	3.14 (4)
Blood pressure regulators	6.3 (8)	Antihistamines	2.36 (3)
Neurological	4.7 (6)	Corticosteroids	2.36 (3)

Regarding the purchased medicines, they were bought by 73 of the respondents (75.3%), and the most common acquisition points were recognized chain pharmacies (n = 49, 80.8%), local pharmacies (n = 23, 31.5%), supermarkets (n = 8, 10.1%), and some other places, which included flea markets, grocery stores, and through online intermediaries (n = 3, 4.1%).

### 3.2 Validation assessment

For qualitative assessment, five experts (two PhD and three master’s degree holders) compiled a self-administered questionnaire and reviewed the scale’s items for grammatical accuracy, wording, avoiding the use of technical terms, item placing, and cultural relevance to the Mexican population.

After reliability analysis, the scale demonstrated acceptable internal consistency reliability with Cronbach’s α at 0.751, in which 25 items were considered. In addition, five dimensions were created and analyzed: socioeconomic dimension (nine items with a Cronbach’s α at 0.921), physical health dimension (four items with a Cronbach’s α at 0.617), cognitive dimension (three items with a Cronbach’s α at 0.446), public health dimension (five items with a Cronbach’s α at 0.508), and safety and quality dimension (seven items with a Cronbach’s α at 0.462).

Regarding the validity of the instrument, construct validation was carried out using exploratory factor analysis (EFA) by principal components, the Promax rotation method with Kaiser normalization; the assumptions of application of the factor analysis were checked with the Kaiser–Meyer–Olkin (KMO) index close to 1 (KMO = 0.728), and Bartlett’s sphericity test was significant at p < 0.001.

### 3.3 Public perception of falsified drugs

When assessing the perception of falsified or counterfeit drugs, the identification of current health alerts for drugs issued by the corresponding regulatory authority (COFEPRIS) was asked, and only 37.1% (33 people out of 89) noticed such a note, whereas 62.9% (56 people out of 89) reported that they were not informed, despite this information being available in social media.

To go deeper into this point, an open question was posed to find out if they personally considered it a relevant and important topic, and the open answers are grouped and described in Table 2, highlighting that 84.3% consider it an important issue, whereas 10.1% answered that they were not sure if this problem would impact them directly or indirectly; meanwhile, 5.6% were not interested in this subject. In this regard, no significant statistical differences were found among gender, those who had a previous diagnosis, or those who recognized health alerts (p > 0.05); meanwhile, a trend but not a difference was observed in those who recently purchased medications and are more aware of the problem compared to those who have not purchased medications (89.0% vs. 11.0%, respectively, p = 0.060).

**TABLE 2:** Categorical answers to the question “Do you think it is an issue that affects you directly or indirectly?”, with the general interpretation for it.

Answer	Why does it affect you directly or indirectly?
Yes	“For their profession, because they normally consume medicines, because of the poor quality of them and they can falsify the ones they consume, because of the concern that their family may buy them, a drug without the characteristics does not work as it should, because of health, because it can affect your body, these carry health risks, because it can be done in commonly used drugs and affects many people, because they must be controlled” The answer in general is because falsified drugs produce a harmful effect on health directly and indirectly, and a falsified drug that does not contain the same components as the original does not have the same effect, and as they buy medicines frequently, they would not want to have health problems.
No	“If drugs are bought in pharmacies, it is expected that this effect will not occur, they feel that they are giving bad publicity to pharmaceutical companies and market leaders, they do not buy medicines as frequently, because I buy medicines in recognized pharmacies, because they are generic medicines that carry the same formula as patent medicines” The answer in general is that it is on the part where they do not buy drugs frequently, or they buy them from recognized pharmacies.
I do not know	“Because they do not take drugs daily,” “they do not frequently consume drugs,” “they do not know about the issue,” “they do not believe that it is a serious issue” The responses in general are observed around the lack of information about the situation and that they do not take drugs frequently.

**FIGURE 1:**
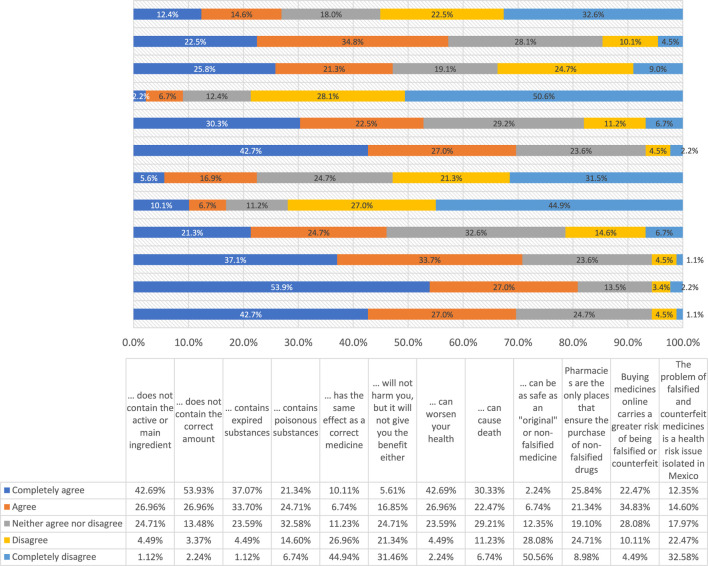
Perception of the risk caused by falsified drugs, report of participants.

Furthermore, regarding the questions focused on their awareness of the nature and implications of falsified drugs in terms of health, what stands out most about these answers is that a considerable percentage of people (50.6%) consider that they would not be able to recognize a fake drug from an original one, with 28.1% suggested being able to differentiate them, and 21.3% did not recognize being able or unable to differentiate them. The complete answers for each item in this section are described in Table 3 and can also be seen in Supplementary Appendix A1 in Figure 1. Although the majority considered that the falsified drugs did not contain an active ingredient or the correct amount of it (69.7% and 80.9%, respectively), 70.8% thought that these medicines are those that contain expired substance(s); meanwhile, 46.1% alluded to the presence of a poisonous substance. Consistent with these perceptions, 71.9% of the respondents deemed that these medications would not have the same efficacy as the patent medicines or will not provide the same benefit despite not causing any harm (22.4%).

**TABLE 3:** Knowledge of falsified and counterfeit medicines and their possible impact on health.

A falsified or counterfeit medicine…	Completely agree (%)	Agree (%)	Neither agree nor disagree (%)	Disagree (%)	Completely disagree (%)
… does not contain the active or main ingredient	42.7	27.0	24.7	4.5	1.1
… does not contain the correct amount	53.9	27.0	13.5	3.4	2.2
… contains expired substances	37.1	33.7	23.6	4.5	1.1
… contains poisonous substances	21.3	24.7	32.6	14.6	6.7
… has the same effect as a correct medicine	10.1	6.7	11.2	27.0	44.9
… will not harm you, but it will not give you the benefit either	5.6	16.9	24.7	21.3	31.5
… can worsen your health	42.7	27.0	23.6	4.5	2.2
… can cause death	30.3	22.5	29.2	11.2	6.7
… can be as safe as an “original” or non-falsified medicine	2.2	6.7	12.4	28.1	50.6
Pharmacies are the only places that ensure the purchase of non-falsified drugs	25.8	21.3	19.1	24.7	9.0
Buying medicines online carries a greater risk of being falsified or counterfeit	22.5	34.8	28.1	10.1	4.5
The problem of falsified and counterfeit medicines is a health risk issue isolated in Mexico	12.4	14.6	18.0	22.5	32.6

Regarding their possible impact on health, most of participants, 78.6%, were aware that these medicines are not as safe as the “original” ones; likewise, 69.7% agreed that falsified or counterfeit medicine could worsen their health and even cause death (52.8%), suggesting that the majority are conscious of the possible negative impact if they consume them. When comparing all these responses according to the sociodemographic and health variables, the recent purchase of medications, and the identification of health alerts, the distribution was similar, with no differences being observed.

Despite 27.0% of the respondents considering falsified and counterfeit medicines to be a health risk isolated in México, 47.2% of the respondents trust that only pharmacies are safe places to acquire medicines, but 33.7% claimed the opposite, whereas 57.3% recognized that the risk of finding these unregulated products in online stores is increasing. Moreover, 83.1% stated that they would not buy from unauthorized points of sale for security reasons, consistently and independently of the respondent’s profile (p < 0.05), suggesting come concern or awareness regarding the care of their health.

**FIGURE 2:**
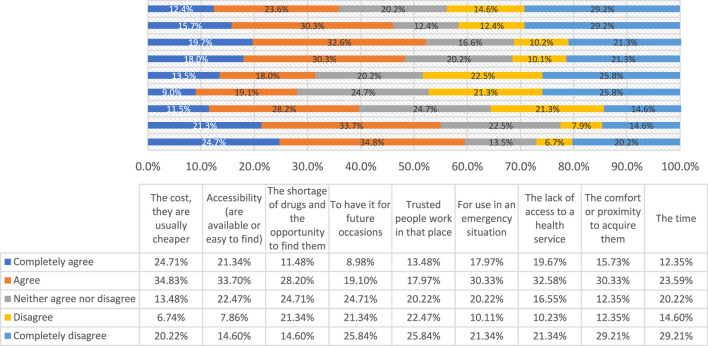
Factors that would influence the purchase of drugs at unauthorized points of sale such as flea markets, markets and grocery stores.

However, when analyzing the responses aimed at knowing the possible factors that could influence the intention and purchase of medicines at unauthorized points of sale if necessary, 10.11% suggested they would buy them for several reasons, as described in Table 4 and in Supplementary Appendix A1, Figure 2, highlighting the responses to a positive purchase intention based on their lower cost (59.5%), the lack of access to a public or private health service (52.3%), accessibility (55.0%), or shortage (39.7%). To a lesser extent, factors related to an emergency of use or the proximity of these points of sale do not appear to influence this decision. Logistic regression was performed in order to identify if these factors could explain the intention of purchase at unauthorized spots and no economic, accessibility, shortage, time, nor forecast factor appeared to be associated.

**TABLE 4:** Factors that would influence the purchase of drugs at unauthorized points of sale, such as flea markets, markets, and grocery stores.

If necessary, I would acquire medicine in flea markets, grocery stores, or some other places that are not a pharmacy or the specific areas in chain supermarkets because…	Completely agree (%)	Agree (%)	Neither agree nor disagree (%)	Disagree (%)	Completely disagree (%)
The cost, they are usually cheaper	24.7	34.8	13.5	6.7	20.2
Accessibility (are available or easy to find)	21.3	33.7	22.5	7.9	14.6
The shortage of drugs and the opportunity to find them	11.5	28.2	24.7	21.3	14.6
To have it for future occasions	9.0	19.1	24.7	21.3	25.8
Trusted people work in that place	13.5	18.0	20.2	22.5	25.8
For use in an emergency situation	18.0	30.3	20.2	10.1	21.3
The lack of access to a health service	19.7	32.6	16.6	10.2	21.3
The comfort or proximity to acquire them	15.7	30.3	12.4	12.4	29.2
The time	12.4	23.6	20.2	14.6	29.2

## 4 Discussion

Notwithstanding the presence of falsified and counterfeit medicines in the market, mostly in unauthorized points of sales, being considered a substantial challenge in the realm of public health, it is an issue not exclusive to low- and middle-income regions, where it has been suggested that one in 10 medical products is substandard or counterfeit (World Health Organization, 2017). Despite this, in Mexico, it continues to be a topic that has been addressed little; however, the existence of counterfeits of medications that are expensive or difficult to obtain is a longstanding and well-described problem, mostly in touristic regions (Friedman et al., 2023), and despite a constant trend of increased trafficking being reported since decades before the outbreak of the COVID-19 pandemic (A Ziavrou et al., 2022).

According to the Pharmaceutical Security Institute, pharmaceutical crime incidents have increased worldwide by 4% in 2023, placing Latin America as the fifth affected region in the order of frequency (Pharmaceutical Security Institute, 2024), and Mexico was the sixth largest country in the world in illegal drug trafficking and trade until 2020, according to reports from the Attorney General’s Office and the National Association of Pharmacies in Mexico. According to the National Chamber of the Pharmaceutical Industry in Mexico, approximately 8 million people have bought stolen or counterfeit medicines (Macip Martínez, 2023). These figures do not necessarily show that low incident reports are not unaffected because of a lower risk of counterfeiting, falsified, or illegal medicine-related incidents but rather because of under detection, the lack of law enforcement priorities or funding, and even an inadequate structure to assess and combat this issue.

This is further complicated by the lack of agreement on a standard definition used for sub-standard, falsified, fake, spurious, or those which infringe local patents and hence are “counterfeit” medicines that can be found on the market, which hinder discussions on this problem of potential risk beyond the individual level and complicate how these issues have been addressed globally (McManus and Naughton, 2020; Sirrs, 2023).

Currently, drugs or medicines are considered as follows: (a) falsified, meaning that they intent to resemble real ones, either because they have been deliberately mislabeled in their name or identity and source of composition, are failing to meet recognized standards of quality due to an incorrect formulation (lower or higher doses than indicated on the label or different ingredients or with no active substances at all), or because of the use of adulterated or expired substances; (b) counterfeit because they somehow infringe on local patents (Świeczkowski et al., 2020) and regulations as a result of improper manufacture, storage, or distribution, or as the outcome of repacking, theft, or resale of approved drugs (A Ziavrou et al., 2022), resulting in a lack of consensus on the terms or definitions, and even the indiscriminate use of synonyms (Ahmed et al., 2022).

Thus, in this study, both terms were used in the applied questionnaire for several reasons: first, as the definition of irregular medicines handled by the agency in charge, COFEPRIS, which includes falsified medicines, those without sanitary registration, and those that are fractionated, expired, and adulterated, which represent risks to the health of the patients, as they could be made with contaminated, toxic substances or have lost their effectiveness due to incorrect storage or transport (Comisión de Fomento Sanitario, 2022), as well as the definition given by the WHO and national regulation, which includes falsified medicines as medical products that deliberately or fraudulently misrepresent their identity, composition or origin, also mentions them as substandard quality, defining them as authorized medical products that do not meet either quality standards or their specifications, or both (World Health Organization, 2024; Alvarez, 2024; Diario Oficial de la Federación, 2024), and also as a result of querying the experts for several reasons; as the objective was not to describe falsified medicines in great detail, to avoid technicalities, and more importantly, to include the wide range of medicines, besides the original, to which the consumer is exposed.

A reliability analysis, using the Cronbach’s alpha coefficient, indicated that the 25 items of the test have an internal consistency of 75.1%. This means the items are correlated and consistently measure the construct. In our results, the participants represent a young and relatively healthy population in accordance with the age structure of the population of Mexico City, which has changed over the last 10 years, and nowadays, the proportion of people aged 25 years and older has increased by 1.3% for those in the working age (INEGI, 2021), and those who, despite reporting a low prevalence of diagnosed diseases, referred to the purchase of medicines on a regular basis.

The medications that were most frequently acquired were analgesics, gastrointestinal agents, antibiotics, anti-flu drugs, and hormonal and blood pressure regulators, in accordance with what has been suggested to be the most encountered types of falsified medicines such as antibiotics and lifestyle medicines, with the exception of anti-malarials (World Health Organization, 2017), suggesting that demands, region, shortages, cost, and the ease of falsifying explain these variations (Bakker-’t Hart et al., 2021). In Mexico, the last health alerts made by COFEPRIS encompass a wide range of medicines, including both commonly used medications and even some specialized for the management of oncological diseases and hormonal disorders, such as levothyroxine sodium, somatropin, and capecitabine (Secretaría de Salud, 2024).

Furthermore, although governments and national drug regulatory agencies, COFEPRIS in Mexico, enshrine particular terms in the legislation and regulatory practices, including efforts aimed at issuing and disseminating alerts addressed to health professionals and the general population regarding the probable presence of falsified or illegal marketing of medicines and their health consequences (Comisión de Fomento Sanitario, 2022; Macip Martínez, 2023; Cordell et al., 1996; Lee et al., 2017; Sanitarios and contra, 2024), our data suggest that most of them are not well informed of the constant alerts regarding the presence of these drugs on the market despite being disseminated through social media and news, as reported in some other populations (Ofori-Parku and Park, 2022; OECD/EUIPO, 2020; Fittler et al., 2018).

In addition, although most participants in this study were aware that these products are not as safe as the original or non-falsified ones and that their intake could worsen their health by prolonging their illnesses and increasing the likelihood of treatment failure (Bottoni and Caroli, 2019; Ozawa et al., 2018; World Health Organization, 2010b), they recognize that they are not able to differentiate an original medicine from a falsified or counterfeit one based on the appearance (El-Dahiyat et al., 2021), which is also complicated even for activists, regulators, and pharmaceutical firms (Kelesidis et al., 2007; Alfadl et al., 2013).

This highlights the urgent need to disseminate reliable information to the public through accessible channels. The goal is to equip individuals with tools to identify suspicious medicines. This includes educating them on recognizing risk outlets and providing critical information for visually inspecting packaging materials. Key indicators of suspicious products include misrepresentation of identity or composition (e.g., mislabeling), and damaged or illegible primary or secondary packaging (Feeney et al., 2024; Comisión de Fomento Sanitario, 2022).

Providing this information in an updated and recurring manner will promote public responsibility and raise expectations regarding the safety of medical products and services (Comisión Federal para la Protección contra Riesgos Sanitarios, 2024; Valente de Almeida et al., 2024; Genovese et al., 2017). This effort should be coordinated with regulatory institutions, which are actively working to improve medicine authentication technology, enhance website verification approaches, and develop new detection methods (Kelesidis et al., 2007; Roth et al., 2018).

In this regard, several factors have been proposed to influence the purchase of illicit medicines, with access and cost being significant contributors (Bian and Moutinho, 2009). Our findings indicate that a primary reason for acquiring these medicines from unauthorized sources, when necessary, is the lack of access to formal healthcare services. This persists despite a notable increase in the insured population in Mexico City over the last decade, from 63.8% to 72.6% (INEGI, 2021), highlighting a continued gap in health coverage.

The illicit medicine market is seen as an unregulated response to the escalating cost of both private and public healthcare, aiming to ensure product availability and distribution. This situation is further exacerbated by drug shortages (Shukar et al., 2021), the demand for cheaper medicines, pervasive corruption, the proliferation of illicit points of sale, inadequate regulatory enforcement, and the absence of advanced technologies for market analysis and monitoring of available medicines (Ofori-Parku and Park, 2022; Feeney et al., 2024).

Although current literature offers valuable information on falsified or counterfeit medications, research gaps remain. To the best of our knowledge, this is the first study that provides an approximation to assess the awareness and knowledge of counterfeit and falsified medicines among a group of apparently young healthy people who are at the risk of acquiring these types of products when dealing with treatments for acute events.

One of the main limitations of this study is the homogeneous and apparently healthy and young sample of individuals, who, despite not having a chronic disease diagnosis, are exposed to the use of medications. Although the findings cannot be extrapolated, the results reported in this study could serve to delve deeper into the issue and analyze possible differences in perception and awareness among those who are chronically exposed to medications and who probably have different purchasing habits or obtain medications through health services, insurance agencies, or established points of purchase.

As we do not claim to have completely captured the perception and our data came from a single region of Mexico, the results should be considered only for hypothesis generation and limited. Our expectation is that our exploratory approach and results will be useful, with adaptations, to detail for the future of such studies in other settings or specific populations to outline public health communication and advocacy efforts to improve consumer decision-making.

## 5 Conclusion

The falsified and counterfeit medicines’ market is a substantial and understudied issue affecting not only individual or collective health. These findings shed light on consumers’ knowledge, risk perceptions, and purchase intentions, which can lead to avenues for future research that can contribute to a more robust understanding of this critical global health issue in line with the available conceptual frameworks and, therefore, can fill the gap and ultimately produce or enforce public policies to promote the rational use of medicines, as well as emphasize the need for more effective post-marketing surveillance strategies to potentially deter the purchasing of falsified or counterfeit medicines.

## Data Availability

The raw data supporting the conclusions of this article will be made available by the authors, without undue reservation.

## Abbreviations

DOAJ, directory of open access journals; TLA, three letter acronym; LD, linear dichroism.

## References

[B1] A Ziavrou K. S. Noguera S. Boumba V. A. (2022). Trends in counterfeit drugs and pharmaceuticals before and during COVID-19 pandemic. Forensic Sci. Int. 338, 111382. 10.1016/j.forsciint.2022.111382 35882074 PMC9277998

[B2] Ahmed J. Modica de Mohac L. Mackey T. Raimi-Abraham B. (2022). A critical review on the availability of substandard and falsified medicines online: incidence, challenges and perspectives. J. Med. access 6, 23992026221074548. 10.1177/23992026221074548 36204527 PMC9413502

[B3] Alfadl A. A. Hassali M. A. Ibrahim M. I. M. (2013). Counterfeit drug demand: perceptions of policy makers and community pharmacists in Sudan. Res. Soc. Adm. Pharm. 9 (3), 302–310. 10.1016/j.sapharm.2012.05.002 22835708

[B4] Alvarez R. J. (2024). Ley general de salud, 53.

[B6] Bakker-’t Hart I. M. E. Ohana D. Venhuis B. J. (2021). Current challenges in the detection and analysis of falsified medicines. J. Pharm. Biomed. Analysis 197, 113948. 10.1016/j.jpba.2021.113948 33582458

[B7] Bian X. Moutinho L. (2009). An investigation of Determinants of counterfeit purchase Consideration. J. Bus. Res. 62 (3), 368–378. 10.1016/j.jbusres.2008.05.012

[B8] Bottoni P. Caroli S. (2019). Fake pharmaceuticals: a review of current analytical approaches. Microchem. J. 149, 104053. 10.1016/j.microc.2019.104053

[B9] Comisión de Fomento Sanitario (2019). Manual Para La Identificación de Medicamentos Falsificados.

[B10] Comisión Federal para la Protección contra Riesgos Sanitarios (2024). Comisión Federal para la Protección contra Riesgos Sanitarios. Podcast Cofepris. Available online at: https://open.spotify.com/show/1rZZtuI1DG7UK9KJn4UbES (Accessed 2024-November-26).

[B11] Cordell V. V. Wongtada N. Kieschnick R. L. (1996). Counterfeit purchase intentions: role of lawfulness attitudes and product Traits as Determinants. J. Bus. Res. 35 (1), 41–53. 10.1016/0148-2963(95)00009-7

[B12] Diario Oficial de la Federación (2024). NORMA Oficial Mexicana NOM-059-SSA1-2015, Buenas prácticas de fabricación de medicamentos. Available online at: https://dof.gob.mx/nota_detalle.php?codigo=5424575&fecha=05/02/2016#gsc.tab=0 (Accessed November 26, 2024).

[B13] El-Dahiyat F. Fahelelbom K. M. S. Jairoun A. A. Al-Hemyari S. S. (2021). Combatting substandard and falsified medicines: public awareness and identification of counterfeit medications. Front. Public Health 9, 754279. 10.3389/fpubh.2021.754279 34765583 PMC8575769

[B14] Feeney A. J. Goad J. A. Flaherty G. T. (2024). Global perspective of the risks of falsified and counterfeit medicines: a critical review of the literature. Travel Med. Infect. Dis. 61, 102758. 10.1016/J.TMAID.2024.102758 39218049

[B15] Fittler A. Vida R. G. Káplár M. Botz L. (2018). Consumers turning to the internet pharmacy market: cross-sectional study on the frequency and attitudes of Hungarian patients purchasing medications online. J. Med. Internet Res. 20 (8), e11115. 10.2196/11115 30135053 PMC6125612

[B16] Friedman J. Godvin M. Molina C. Romero R. Borquez A. Avra T. (2023). Fentanyl, heroin, and Methamphetamine-based counterfeit Pills sold at Tourist-Oriented pharmacies in Mexico: an Ethnographic and drug checking study. Drug Alcohol Dependence 249, 110819. 10.1016/j.drugalcdep.2023.110819 37348270 PMC10368172

[B17] Genovese U. Del Sordo S. Pravettoni G. Akulin I. M. Zoja R. Casali M. (2017). A new Paradigm on health care Accountability to improve the quality of the system: Four Parameters to Achieve individual and collective Accountability. J. Glob. Health 7 (1), 010301. 10.7189/jogh.07.010301 28567274 PMC5441445

[B18] González L. M. (2024). Cada vez hay más medicamentos falsos en México: Cofepris - Amexi. Available online at: https://amexi.com.mx/nacional/cada-vez-hay-mas-medicamentos-falsos-en-mexico-cofepris/(Accessed October 22, 2024).

[B19] INEGI (2021). EN la CIUDAD de méxico SOMOS 9 209 944 habitantes, 1.

[B20] Kelesidis T. Kelesidis I. Rafailidis P. I. Falagas M. E. (2007). Counterfeit or substandard antimicrobial drugs: a review of the scientific Evidence. J. Antimicrob. Chemother. 60 (2), 214–236. 10.1093/jac/dkm109 17550892

[B21] Lee K. S. Yee S. M. Zaidi S. T. R. Patel R. P. Yang Q. Al-Worafi Y. M. (2017). Combating sale of counterfeit and falsified medicines online: a losing Battle. Front. Pharmacol. 8, 268. 10.3389/fphar.2017.00268 28559845 PMC5432535

[B22] Macip Martínez I. (2023). Ciencia cofepris, 46.

[B23] McManus D. Naughton B. D. (2020). A systematic review of substandard, falsified, unlicensed and Unregistered medicine sampling studies: a Focus on context, prevalence, and quality. BMJ Glob. Health 5 (8), e002393. 10.1136/bmjgh-2020-002393 32859648 PMC7454198

[B24] Meraz A. M. (2018). Sexto Venta Med. Ilegal 12.

[B5] OECD/EUIPO (2020). Trade in counterfeit pharmaceutical products, Illicit Trade. Paris: OECD Publishing. 10.1787/a7c7e054-en

[B25] Ofori-Parku S. S. Park S. E. I. (2022). I (Don't) want to consume counterfeit medicines: exploratory study on the antecedents of consumer attitudes toward counterfeit medicines. BMC Public Health 22 (1), 1094. 10.1186/s12889-022-13529-7 35650557 PMC9158175

[B26] Ozawa S. Evans D. R. Bessias S. Haynie D. G. Yemeke T. T. Laing S. K. (2018). Prevalence and estimated economic burden of substandard and falsified medicines in low- and middle-income countries: a systematic review and meta-analysis. JAMA Netw. Open 1 (4), e181662. 10.1001/jamanetworkopen.2018.1662 30646106 PMC6324280

[B27] Pharmaceutical Security Institute (2024). Geographic distribution. PSI. Available online at: https://www.psi-inc.org/geographic-distribution (Accessed November 02, 2024).

[B28] Protección Contra Riesgos Sanitarios (2013). Programa de Acción Específico.

[B29] Rodríguez A. (2024). Robo y falsificación de medicamentos “se dispara” más de 300% en 2022. Available online at: https://www.elfinanciero.com.mx/empresas/2023/05/31/robo-y-falsificacion-de-medicamentos-se-dispara-mas-de-300-en-2022/(Accessed October 18, 2024).

[B30] Roth L. Nalim A. Turesson B. Krech L. (2018). Global landscape assessment of screening technologies for medicine quality assurance: stakeholder perceptions and practices from ten countries. Glob. Health 14 (1), 43. 10.1186/s12992-018-0360-y 29695278 PMC5922304

[B31] Sanitarios C. F. contra R. (2024). “para la P,” in Cofepris alerta sobre falsificación de 7 medicamentos y venta ilegal de fármaco no autorizado. Available online at: http://www.gob.mx/cofepris/es/articulos/cofepris-alerta-sobre-falsificacion-de-7-medicamentos-y-venta-ilegal-de-farmaco-no-autorizado?idiom=es (Accessed November 12, 2024).

[B32] Secretaría de Salud (2024). Alertas sanitarias. Instituto de Salud del Estado de México. Available online at: https://salud.edomex.gob.mx/isem/alertas_sanitarias (Accessed November 19, 2024).

[B33] Secretaria de Salud del Estado de Nuevo León (2024). Comunicados y alertas sanitarias. Available online at: https://saludnl.gob.mx/regulacion-sanitaria/index.php/comunicados-y-alertas-sanitarias-2024/(Accessed October 16, 2024).

[B34] Shukar S. Zahoor F. Hayat K. Saeed A. Gillani A. H. Omer S. (2021). Drug shortage: causes, impact, and mitigation strategies. Front. Pharmacol. 12, 693426. 10.3389/fphar.2021.693426 34305603 PMC8299364

[B35] Sirrs C. (2023). Fluid fakes, contested counterfeits: the world health Organization’s Engagement with fake drugs, 1948–2017. Med. Anthropol. Theory 10, 1–29. 10.17157/mat.10.3.7234 38660628

[B36] Świeczkowski D. Zdanowski S. Merks P. Szarpak Ł. Vaillancourt R. Jaguszewski M. (2020). The Plague of Unexpected drug Recalls and the pandemic of falsified medications in Cardiovascular medicine as a threat to patient safety and global public health: a Brief review. Cardiol. J. 29, 133–139. 10.5603/CJ.a2020.0168 33346374 PMC8890415

[B37] Valente de Almeida S. Hauck K. Njenga S. Nugrahani Y. Rahmawati A. Mawaddati R. (2024). Value for Money of medicine sampling and quality testing: evidence from Indonesia. BMJ Glob. Health 9, e015402. 10.1136/bmjgh-2024-015402 39313254 PMC11429347

[B38] World Health Organization (2024). Productos médicos de calidad subestándar y falsificados. Available online at: https://www.who.int/es/news-room/fact-sheets/detail/substandard-and-falsified-medical-products (Accessed November 19, 2024).

[B39] World Health Organization (2010a). Falsificación de productos médicos, 4–6.

[B40] World Health Organization (2010b). Falsificación de productos médicos.

[B41] World Health Organization (2017). WHO global surveillance and monitoring system for substandard and falsified medical products. Geneva: World Health Organization.

