# Supplementary material for: Perception of falsified and counterfeit medicines among adults living in Mexico City and the metropolitan area: an underappreciated health risk
Source: Front Pharmacol. 2025 Aug 29;16:1654822. doi: 10.3389/fphar.2025.1654822 (PMC12426161; doi:10.3389/fphar.2025.1654822)
Supplement: Supplementary file 1 [file DataSheet1.pdf]

Appendix A

Table 1A. Drugs reported as falsified by COFEPRIS in the year 2024 until September.

Date	Falsified drug	Presentation
September 24, 2024	Trayenta (linagliptin)	5 mg
	Trayenta Duo (linagliptin/metformin)	2.5 mg/850 mg
September 12, 2024	Beneflur (fludarabine)	10 mg
September 09, 2024	Cafiaspirina (acetylsalicylic acid/caffeine)	500 mg/30 mg
	Aspirina (acetylsalicylic acid)	500 mg
	Aspirina protec (acetylsalicylic acid)	1000 mg
	Desenfriol D (chlorpheniramine/phenylephrine/paracetamol)	2 mg/5 mg/500 mg
	Desenfriol-ito plus (chlorphenamine/phenylephrine/paracetamol)	1 mg/2.5 mg/80 mg
	Tabcin noche (paracetamol, dextromethorphan hydrobromide/doxylamine succinate/phenylephrine hydrochloride)	-
August 19, 2024	Zyrtec (cetirizine)	10 mg/1mL
August 09, 2024	Moveloc (propofol)	1% injectable emulsion
August 07, 2024	Tiaminal B12 50,000 (cyanocobalamin/thiamine/lidocaine)	-
	Octagam 5% (intravenous human normal immunoglobulin)	-
July 09, 2024	Cialis (tadalafil)	20 mg
July 02, 2024	Eutirox (levothyroxine sodium)	100 mg
June 25, 2024	Saizen (somatropin)	12 mg/1,5mL
	Saizen (somatropin)	20 mg
June 21, 2024	Erbitux (cetuximab)	5 mg/mL

June 20, 2024	Xeloda (capecitabine)	500 mg
June 17, 2024	Lumigan (Bimatoprost)	0.03% solution
June 07, 2024	Dolo-Neurobión Forte DCS (B complex (thiamine, pyridoxine, cyanocobalamin) /lidocaine/diclofenac sodium)	Injectable solution
May 28, 2024	Pomada Green MARVEL	-
April 23, 2024	Kadcyla (trastuzumab emtansine)	100 mg
	Stribild (elvitegravir, cobicistat, emtricitabine, tenofovir)	150 mg/150 mg/200 mg/300 mg
	Anesket (ketamine)	50 mg/mL and 100 mg/mL
April 17, 2024	Restylane Kysse (lidocaine gel)	-
February 22, 2024	Krytantek Ofteno (dorzolamide/timolol/brimonidine)	5 mL
	Krytantek Ofteno MMNN INT (prednisolone)	3 mL
January 08, 2024	Forxiga (dapagliflozin)	10 mg
	Lakesia (ciclopirox)	Lacquer/ 8% solution
	Alistint (alprostadil)	20 µg/mL and 500 µg/mL
	Tykerb (lapatinib)	250 mg
	Dupixent (dupilumab)	300 mg/2mL

This is a provisional file, not the final typeset article
